# Cell-Type-Specific Heat-Induced Changes in the Proteomes of Pollen Mother Cells and Microspores Provide New Insights into Tomato Pollen Production Under Elevated Temperature

**DOI:** 10.3390/proteomes13020013

**Published:** 2025-03-25

**Authors:** Priya Thapa, Jun Guo, Kajol Pradhan, Dibya Thapa, Sudhakar Madhavarapu, Jing Zou, Jesse Potts, Hui Li, Joshua O’Hair, Chen Wang, Suping Zhou, Yong Yang, Tara Fish, Theodore W. Thannhauser

**Affiliations:** 1 Department of Agricultural Sciences and Engineering, College of Agriculture, Tennessee State University, 3500 John Merritt Blvd, Nashville, TN 37209, USA; thapapriya.pt@gmail.com (P.T.); jguo@my.tnstate.edu (J.G.); dmagar@my.tnstate.edu (D.T.); msudhaka@my.tnstate.edu (S.M.); jzou@my.tnstate.edu (J.Z.); jpotts3@ufl.edu (J.P.); johair@tnstate.edu (J.O.);; 2 R.W. Holley Center for Agriculture and Health, USDA-ARS, Cornell University, Ithaca, NY 14853, USAtlf26@cornell.edu (T.F.)

**Keywords:** pollen mother cells, free microspores, meiosis, protein translation, pollen wall, thermotolerance, single-cell-type proteomics, *Solanum lycopersicum*

## Abstract

Background: Tomatoes are self-pollinating plants, and successful fruit set depends on the production of functional pollen within the same flower. Our previous studies have shown that the ‘Black Vernissage’ tomato variety exhibits greater resilience to heat stress in terms of pollen productivity compared to the ‘Micro-Tom’ variety. Pollen productivity is determined by meiotic activity during microsporogenesis and the development of free microspores during gametogenesis. This study focused on identifying heat stress (HS)-induced proteomes in pollen mother cells (PMCs) and microspores. Methods: Tomato plants were grown under two temperature conditions: 26 °C (non-heat-treated control) and 37 °C (heat-treated). Homogeneous cell samples of meiotic PMCs (prior to the tetrad stage) and free microspores were collected using laser capture microdissection (LCM). The heat-induced proteomes were identified using tandem mass tag (TMT)–quantitative proteomics analysis. Results: The enrichment of the meiotic cell cycle in PMCs and the pre-mitotic process in free microspores confirmed the correlation between proteome expression and developmental stage. Under HS, PMCs in both tomato varieties were enriched with heat shock proteins (HSPs). However, the ‘Black Vernissage’ variety exhibited a greater diversity of HSP species and a higher level of enrichment compared to the ‘Micro-Tom’ variety. Additionally, several proteins involved in gene expression and protein translation were downregulated in PMCs and microspores of both varieties. In the PMC proteomes, the relative abundance of proteins showed no significant differences between the two varieties under normal conditions, with very few exceptions. However, HS induced significant differential expression both within and between the varieties. More importantly, these heat-induced differentially abundant proteins (DAPs) in PMCs are directly involved in meiotic cell division, including the meiosis-specific protein ASY3 (Solyc01g079080), the cell division protein kinase 2 (Solyc11g070140), COP9 signalosome complex subunit 1 (Solyc01g091650), the kinetochore protein ndc80 (Solyc01g104570), MORC family CW-type zinc finger 3 (Solyc02g084700), and several HSPs that function in protecting the fidelity of the meiotic processes, including the DNAJ chaperone (Solyc04g009770, Solyc05g055160), chaperone protein htpG (Solyc04g081570), and class I and class II HSPs. In the microspores, most of the HS-induced DAPs were consistently observed across both varieties, with only a few proteins showing significant differences between them under heat stress. These HS-induced DAPs include proteases, antioxidant proteins, and proteins related to cell wall remodeling and the generation of pollen exine. Conclusions: HS induced more dynamic proteomic changes in meiotic PMCs compared to microspores, and the inter-varietal differences in the PMC proteomes align with the effects of HS on pollen productivity observed in the two varieties. This research highlights the importance of the cell-type-specific proteomics approach in identifying the molecular mechanisms that are critical for the pollen developmental process under elevated temperature conditions.

## 1. Introduction

Tomato (*Solanum lycopersicum*) is an agriculturally significant crop, with an annual global production of approximately 200 million tons [1]. The global tomato market was valued at USD 195.34 billion in 2023 and is projected to reach USD 294.06 billion by 2032 [2]. In the U.S., tomatoes are the second most consumed vegetable after potatoes [3], contributing a substantial portion of vegetables for human health.

During the reproductive stage, tomatoes require nighttime temperatures below 21 °C and daytime temperatures below 32 °C for healthy flower development and fruit set. Prolonged exposure to mild heat stress (~35 °C) can disrupt pollen production and release, leading to developmental abnormalities [4,5,6]. While tomato plants can tolerate brief (3 h) exposures to extreme heat (40 °C), the reproductive organs of affected flowers often suffer irreversible damage [7]. Both chronic mild heat and short-term extreme heat events are becoming more frequent in major tomato-producing regions worldwide [8,9]. The most popular tomato varieties are heat-sensitive during the flowering and fruit set stages. Tomato plants cultivated on commercial farms as well as in home gardens have been noted to stop bearing fruits during the hot summer season [10,11]. As the number of days exceeding 32 °C increases on a global scale, along with prolonged periods of extreme heat, tomato production will face more severe challenges.

Heat stress (HS) reduces the pollen grain number, viability, and germination rate [12]. Pollen productivity and viability are key indicators of thermotolerance in tomatoes [5]. Pollen grains are developed through two consecutive and differentiated stages: microsporogenesis and gametogenesis. Microsporogenesis begins with the differentiation of pollen mother cells (PMCs) in the anther, which undergo meiosis to produce four haploid unicellular microspores. During gametogenesis, these microspores develop into bi- or tri-nucleate pollen grains through mitosis. These stages are highly sensitive to environmental stresses [13,14,15,16,17]. In tomatoes, meiotic PMCs (meiosis I to meiosis II), unicellular microspores, and the transition to bicellular pollen are particularly vulnerable to HS [18,19]. The heat tolerance mechanisms in each of these specific types of cells can collectively determine the productivity and quality of the pollen grains produced under elevated temperatures.

The response of pollen to elevated temperatures is primarily regulated at the proteome level, with proteins involved in synthesis and degradation playing a central role [20]. Proteomic profiling of five pollen developmental stages—microsporocyte, tetrads, microspores, polarized microspores, and mature pollen—revealed that tomato pollen development is a tightly controlled sequential process [21]. Our previous study showed that the heat-tolerant tomato variety ‘Maxifort’ pollen cells were enriched with heat shock proteins (HSPs) and proteins affecting meiosis fidelity, such as the meiotic serine proteinase and meiotic spindle formation protein mei-1 [22].

Significant differences in HS responses exist among species and cultivars, with heat-tolerant lines often exhibiting higher expression of heat shock factors (Hsfs) and HSPs in pollen and anthers [23,24,25,26]. Heat-tolerant tomato lines also maintain higher levels of starch and sucrose in developing pollen grains compared to sensitive lines [14]. In our study comparing pollen thermotolerance in the tomato varieties ‘Black Vernissage’ and ‘Micro-Tom’, under heat stress at 37 °C for two weeks, we observed a significant reduction in pollen production in both varieties. In ‘Micro-Tom’, the pollen count per flower decreased from 50,240 to 17,539, representing a 65% reduction. In ‘Black Vernissage’, the pollen count declined from 63,085 to 30,360, corresponding to a 52% reduction. The higher pollen count and the lower rate of HS-induced decline in ‘Black Vernissage’ under heat stress suggest greater thermotolerance in pollen production compared to ‘Micro-Tom’ [27].

Pollen production is influenced by the meiotic activity of PMCs during microsporogenesis and the development of free microspores during gametogenesis. In this study, cell-specific quantitative proteomics analyses of meiotic PMCs and unicellular microspores were conducted to identify heat-induced differentially abundant proteins (DAPs) and the underlying molecular mechanisms conferring heat tolerance during pollen development.

## 2. Materials and Methods

### 2.1. Plant Materials and Heat Treatments

Two tomato varieties were used in this study: seeds of ‘Black Vernissage’ were purchased from a local seed store in Nashville, TN, and seeds of ‘Micro-Tom’ were obtained from the C.M. Rick Tomato Genetics Resources Center at the University of California, Davis, CA, USA. Tomato seeds were propagated through self-pollination at the Agricultural Research Station at Tennessee State University, Nashville, TN, USA.

Plants with two true leaves were transplanted into 19 L pots filled with a potting mix consisting of Vegetable Garden soil (Bates Nursery, Nashville, TN, USA) and Tomato-tone (The Espoma Company, Millville, NJ, USA) at a ratio of 10:1 (*v*/*v*). The plants were cultivated in an environmentally controlled plastic greenhouse, with temperatures maintained at 26 °C during the day and 21 °C ± 1° C at night (12/12 h photoperiod), and supplemental LEC lighting provided a light intensity of 700 µmol·m^−2^·s^−1^. The air’s relative humidity was maintained at 65–70% during the day and 70–80% at night and recorded using an EasyLog Data Logger (EL-USB-2, Lascar Electronics, Erie, PA, USA). These growth conditions served as the non-heat-treated control for the tomato plants. Heat treatment was initiated when the tomato plants developed three to five clusters of flowers. The heat-treated plants were transferred to a separate greenhouse, where the temperatures were set to 37 °C/26 °C ± 1 °C (12/12 h), while the light and humidity conditions were the same as in the non-heat-treated control conditions. During the first two weeks of heat treatment, flowers reaching the anthesis stage were manually removed. Samples were collected in the third week of treatment. The experiment included three biological replicates, each consisting of 15 plants, for both the heat-treated and non-heat-treated conditions.

### 2.2. Determination of Flower Bud Growth Process and Correction Between Flower Buds and Pollen Developmental Stages

Flower buds visible to the naked eye were recorded for their growth daily. When the flowers progressed from the smallest bud stage to the first day of anthesis, the buds were collected, and their size was measured as the entire length of the stamen using an Insize vernier caliper [22]. The flower buds were grouped by size, embedded in an optimum-cutting-temperature (OCT) compound, and frozen in liquid nitrogen. Cross-sections of the buds were cut at a thickness of 20 µm using a Leica CM1950 cryostat (Leica, Wetzlar, Germany). The tissue sections were transferred onto pre-coated adhesive slides using the CryoJane Tape-Transfer System (Leica). The structure of the anther locules and cell nuclei was analyzed to determine the pollen developmental stages and their correlation with the sizes of the flower buds.

### 2.3. Collection of Cell-Type-Specific Pollen Cell Samples Using Laser Capture Microdissection

Based on the developmental analysis, tomato ‘Black Vernissage’ flower buds measuring 3.5–4.0 mm in length contained meiotic pollen mother cells (PMCs) and tetrads, while buds measuring 7.0–8.0 mm in length contained unicellular free microspores. For ‘Micro-Tom’, flower buds measuring 2.0–3.0 mm in length contained meiotic PMCs and tetrads, while buds measuring 5.0–6.0 mm in length contained unicellular free microspores. Flower buds within these size ranges were collected separately from the heat-treated and non-heat-treated plants.

Fresh-frozen cross-sections of the flower buds were prepared as described previously [22]. To confirm the pollen developmental stages, one cross-section per bud was stained with 4′,6-diamidino-2-phenylindole (DAPI) (1 µg·mL^−1^) and observed under the Axio Imager M2 microscope (ZEISS). For buds confirmed to be at the desired developmental stages, the middle portions of the anther cone were prepared into 20 µm thick sections. Cells were collected into capture caps using a ZEISS PALM MicroBeam Laser Microdissection system. For each biological replicate, approximately 200,000 cells were collected from 60 flower buds obtained from 15 plants. Three biological replicates were collected for PMCs and microspores each for the two tomato varieties under both heat-treated and non-heat-treated conditions.

### 2.4. Quantitative Proteomics Analysis

Protein extraction. The Pressure Cycling Technology (PCT) method suitable for protein extraction from small amounts of tissue samples was used to extract proteins from the LCM-collected cell samples [22,28]. PCT buffer was prepared to a final concentration of 20 mM 4-(2-hydroxyethyl)piperazine-1-ethane-sulfonic acid (HEPES), 4 M urea, 2% sodium dodecyl sulfate (SDS), 2 mM EDTA, and 1× Halt protease inhibitors (Sigma, St. Louis, MO, USA), pH 8.0. To wash the cells off the LCM capture cap, the PCT buffer was added to cover the surface of the capture resin (45 µL per cap). The cells were collected in the capture tube after centrifugation at 5000 rpm for 5 min at 25 °C. The cell samples were transferred into PCT micro-tubes and sealed with 50 µL pistils, and the entire assembly was then loaded onto a Barocycler 2320 EXT (Pressure Biosciences Inc., Easton, MA, USA). Protein extraction was performed by running a program consisting of 60 repeated cycles between ambient pressure (14.7 psi) and 45 kPsi and 25 °C, and the process was repeated twice. Upon completion of the extraction cycles, the protein extracts were transferred to 1.5 mL low-bind microcentrifuge tubes. After centrifugation at 10,000× *g* for 15 min at 4 °C, the supernatants were transferred to new tubes. The protein concentration was assayed using the Qubit Protein Assay kit on a Qubit 3.0 Fluorometer (Life Technologies, Carlsbad, CA, USA). From approximately 200,000 cells of the three biological replicates, the following protein amounts were extracted: For PMCs, 35–38 µg of proteins from the non-heat-treated samples, and 30–30.5 µg from the heat-treated controls in ‘Black Vernissage’; 24–26 µg from non-heat-treated and 27–29 µg from heat-treated ‘Micro-Tom’. For the microspore cells, 30–31 µg of proteins was extracted from both heat-treated and non-heat-treated ‘Black Vernissage’, and 30–31 µg from non-heat-treated and 28–29 µg from heat-treated ‘Micro-Tom’. Furthermore, the PCT-Barocycler protein extraction method was developed within our group for LCM-collected cell samples as part of the single-cell-type proteomics workflow [18,22].

Trypsin digestion and tandem mass tag (TMT) labeling. For each sample, 18 µg of proteins was taken. After evaporating the moisture under vacuum, the proteins were resolubilized in 41 µL of 1× lysis buffer containing 5% SDS and 50 mM tetraethylammonium bromide (TEAB), pH 8.5. Proteins were reduced using tris (2-carboxyethyl)phosphine (TCEP) (final concentration of 5 mM) followed by alkylation with methyl methanethiosulfonate (MMTS) (final concentration at 20 mM). The protein was acidified by adding phosphoric acid to pH < 1.0 and loaded onto the S-TRAP column (Protifi, Fairport, NY, USA). After washing off SDS and urea, the proteins were digested by loading trypsin at a rate of 4 µg per sample (Promega, Madison, WI, USA). After incubation at 37 °C for 15 h, the digested peptides were eluted from the column followed by evaporation to complete dryness under vacuum.

The tryptic peptides were resuspended in 100 µL of 50 mM TEAB and sonicated for 10 min to ensure complete solubilization. The digested peptides were then labeled using the TMT 16-plex reagent set (Thermo Scientific, Waltham, MA, USA), with 0.25 mg of TMT tag applied to each sample. The PMCs and microspores were analyzed in two separate TMT experiments. For the ‘Black Vernissage’ samples, tags 130N, 129C, and 127N were used to label the three non-heat-treated samples, while tags 133N, 128C, and 132C were used for the three heat-treated samples. For the ‘Micro-Tom’ samples, tags 132N, 131C, and 129N were used for the non-heat-treated samples, and tags 128N, 131N, and 130C were used for the heat-treated samples. After labeling, all samples within the same TMT experiment were pooled and cleaned using an Oasis MCX 1cc extraction cartridge. The peptides were eluted three times with a buffer containing 75% acetonitrile (ACN) and 10% NH4OH. Finally, the labeled peptides were dried under a vacuum and stored at −20 °C until further analysis.

High-pH reverse-phase (hpRP) peptide fractionation. hPH-RP chromatography was carried out using a Dionex UltiMate 3000 HPLC system with the built-in micro-fraction collection option in its autosampler and UV detection (Thermo Scientific, Sunnyvale, CA, USA), as reported previously [29,30,31]. Specifically, the TMT 16-plex-tagged tryptic peptides were reconstituted in buffer A (20 mM ammonium formate pH 9.5 in water) and loaded onto an Xbridge MS C18 column (3.5 µm, 150 mm × 2.1 mm) from Waters (Milford, MA, USA) with buffer A. The peptides were eluted using a gradient from 5 to 45% of buffer B (80% ACN/20% 20 mM NH_4_FA) over 49 min at a flow rate of 200 µL/min. Forty-eight contiguous fractions were collected at 1 min intervals and pooled into a total of 12 fractions based on the UV absorbance at 214 nm with a multiple fraction concatenation strategy [32]. Each of the 12 fractions was dried and reconstituted in 50 µL of 2% ACN/0.5% formic acid (FA) for nano-LC-MS/MS analysis.

Nano-scale reverse-phase chromatography and tandem MS (nanoLC-MS/MS). The nano-LC-MS/MS analysis was carried out using an Orbitrap Eclipse (Thermo-Fisher Scientific, San Jose, CA, USA) mass spectrometer equipped with a nanospray Flex Ion Source coupled to an UltiMate 3000 RSLCnano (Dionex, Sunnyvale, CA, USA). Each reconstituted fraction (5 μL containing ≈ 1 µg of labeled peptides) was injected onto a PepMap C-18 RP nano trap column (5 µm, 100 µm × 20 mm, Dionex) at a 20 μL/min flow rate and separated on a PepMap C-18 RP nano column (2 µm, 75 µm × 25 cm).

Two elution buffers were used: eluent A buffer containing 2% ACN in 0.1% aqueous FA and eluent B containing 95% ACN with 0.1% FA. Prior to each run, the column was equilibrated by running the eluent A buffer. The labeled peptides were eluted in a 120 min gradient of 5% to 32% eluent B buffer. The column was then subjected to an 8 min ramp to 90% eluent B, a 7 min hold, and a 21 min re-equilibration with eluent A buffer prior to the next run. The mass spectrometer (Orbitrap Eclipse) was operated in a setting to specifically select to detect only positively charged ions (“positive ion mode”), with a nanospray voltage of 1.9 kilovolts and a source temperature of 300 °C. External calibration was performed to ensure the mass accuracy of the Fourier Transform (FT), ion trap (IT), and quadrupole mass analyzers. Raw MS data files were acquired following a real-time search (RTS) synchronous precursor selection (SPS) MS^3^ procedure [33].

In this workflow, a 2.5 s “Top Speed” data-dependent CID-MS/MS scan triggers the SPS of 10 product ions for MS^3^ in the FT mass analyzer. In RTS mode, the ITAG2.4 tomato database with 34,633 sequences was imported as a FASTA file for the database search. The search parameters included the following: trypsin as the proteolytic enzyme, TMTpro modifications on lysine and N-terminal amines (∆mass 304.2071), and methylthiol (MMTS) modification of cysteine. The RTS MS^3^ search was configured with a maximum search time of 35 ms. For the MS^3^ scan, the following parameters were selected: mass range of 110–500 m/z, 1.1 m/z for the mass isolation window, and 2.0 *m*/*z* for the MS^2^ isolation window. For peptide quantitation, the MS^3^ with a Full-Width at Half Max (FWHM) resolution of 5000 and a normalized collision energy of 55% was selected. The other parameters included the normalized AGT target of 200% and the maximum injection time set to 120 ms. The dynamic exclusion parameters were set to 1, with a 50 s exclusion duration and a ±10 parts per million (ppm) mass window. All data were collected using an Orbitrap Eclipse (Thermo-Fisher Scientific), controlled by Xcalibur 4.3 software.

Data processing, protein identification, and data analysis. To identify the peptide sequences, the whole set of peptide MS spectra from a single TMT experiment was analyzed using the Sequest HT search algorithm in the Proteome Discoverer 2.5 (PD2.5) software (Thermo-Fisher Scientific). The ITAG2.4 database was used for post-acquisition database searches. The default search settings used for 16-plex TMTpro quantitative processing and protein identification in the PD2.5 search software were selected. The search parameters used were as follows: two missed cleavages for tryptic peptides, fixed cysteine residues modified by MMTS, 16-plex TMT with fixed modifications on lysine and N-terminal amines, and variable modifications including methionine oxidation, deamidation of asparagine/glutamine residues, and protein N-terminal acetylation. The peptide mass tolerance and fragment mass tolerance values were set to 10 ppm for the MS survey scan, 0.6 Da for the MS^2^ scan, and 20 ppm for the MS^3^ scan. All the identified peptides were filtered using the Percolator algorithm at a maximum 1% false discovery rate (FDR) to report peptides with a high confidence level and a mass accuracy ≤ 5 ppm. Raw *p*-values were adjusted by the Benjamini–Hochberg method [34] to control the FDR.

The TMT16-plex quantification method within PD 2.5 was used to calculate the reporter ion abundances from MS^3^ spectra. The “Total Peptide Amount Normalization Method” contained within the PD 2.5 software was used to normalize protein abundances across samples. Correction for isotopic impurities was applied. Only reporter ions meeting the criteria of a co-isolation threshold of 50% and a reporter ion signal-to-noise (S/N) (intensity) threshold of ≥10:1 were included in the quantitative analyses. Both unique and razor (shared) peptide intensities were calculated and reported. The abundance of any quantified protein was determined by summing the intensities of the MS^3^ reporter ions of its unique constituent peptides.

### 2.5. Identification of Differentially Abundant Proteins (DAPs)

Proteins quantified with two or more unique peptides were included for quantitative analysis. For each protein, the ratio of the normalized reporter ion intensity from the three replicates of each sample type within a comparison pair was converted into log (2) fold change values. The log (2) fold change values of all the identified proteins within a proteome were fitted to a normal distribution [22] to obtain the standard deviation (SD). The raw *p*-values were determined using a two-tailed Student’s *t*-test with unequal variance and then adjusted for FDR by applying the Benjamini–Hochberg method [34] using SAS version 9.0 software (SAS Inc., Cary, NC, USA). Differentially abundant proteins (DAPs) were identified using the criteria of a log (2) fold change greater than two standard deviations (±2 SDs) with FDR-adjusted *p* < 0.05.

### 2.6. Functional Analysis of Differentially Abundant Proteins (DAPs)

Protein–protein interaction networks were constructed using STRING (confidence score ≥ 0.400) and visualized in Cytoscape 3.7.1. Functional enrichment analysis was performed using STRING (v11.5). Literature searches were conducted to elucidate the roles of DAPs in pollen developmental processes under HS conditions.

## 3. Results and Analysis

### 3.1. Identification of Cell-Type-Specific Proteomes in PMCs and Microspores

**Figure 1:**
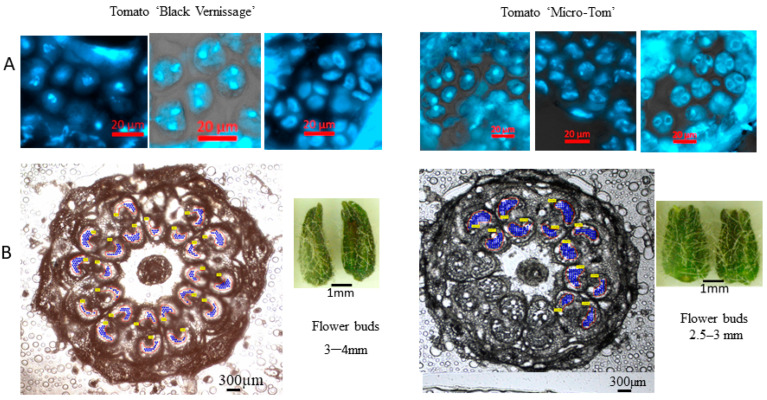
Collection of pollen mother cells using laser capture microdissection. (**A**) Confirmation of pollen mother cell developmental stages stained with DAPI and observed under a Zeiss Axio Imager. Pollen mother cells at meiosis I-II, prior to the tetrad stage, at 400× magnification. (**B**) Images of pollen mother cells collected using a Zeiss PALM MicroBeam under 5× objective magnification.

**Figure 2:**
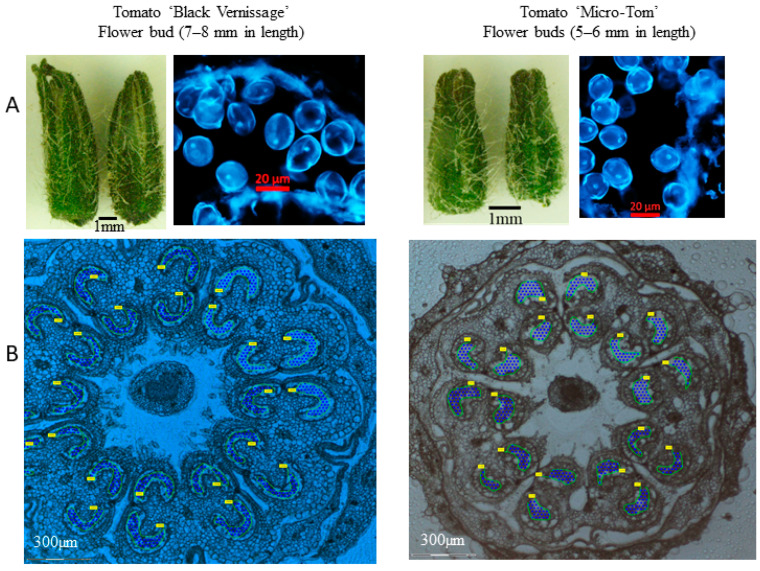
Collection of microspores using laser capture microdissection. (**A**) Confirmation of the developmental stages of microspores stained with DAPI and observed under a Zeiss Axio Imager at 400× magnification; (**B**) Images of microspore collection using a Zeiss PALM MicroBeam under 5× objective magnification.

Tomato flower buds containing meiotic pollen mother cells (PMCs) and microspores were collected from both heat-treated and non-heat-treated plants. Each flower bud was cut into 8–10 sections, with 1 cross-section stained with DAPI and examined under a ZEISS microscope to confirm the pollen developmental stage (Figure 1 and Figure 2). The remaining sections were used for cell collection using LCM. For each biological replicate (representing an individual sample), approximately 200,000 cells were collected.

**Figure 3:**
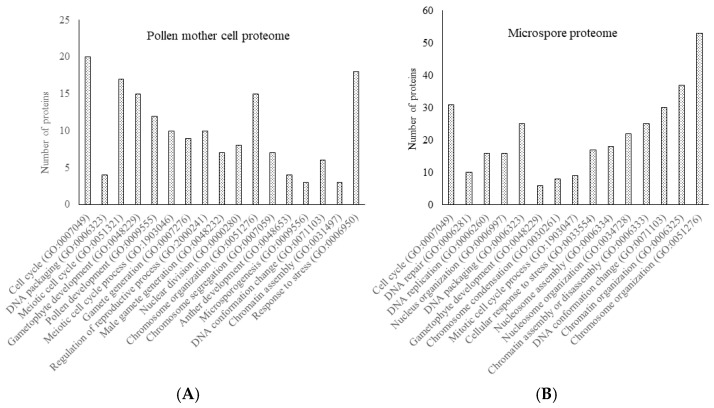
The enriched biological processes in the cell-type-specific proteomes confirm that the quantified proteomes represent the pollen mother cells (PMCs) and microspores. The biological processes were constructed by searching the tomato proteins against the version 11.5 STRING database of *Solanum lycopersicum*, at a false discovery rate < 0.05. Data are provided in Supplementary Material Tables S2 and S4. (**A**) The enriched biological processes from PMC proteomes; (**B**) The enriched biological processes from microspore cells.

TMT proteomics analysis identified 3125 proteins in the PMCs (Supplementary Material Table S1). Upon analyzing the PMC proteomes in STRING, 96 proteins were found to be associated with reproductive processes. Further analysis in the same database revealed that these 96 proteins were involved in biological processes related to the meiotic stages of the pollen mother cells. These include male gamete generation, meiosis, meiotic cell cycle process, chromosome organization involved in the meiotic cell cycle, and response to stress (Figure 3A; Supplementary Material Table S2). From the microspore proteomes, 2642 proteins were quantified (Supplementary Material Table S3). Proteins enriched into nuclei activities are related to pre-mitotic activities including DNA replication, DNA packaging, chromatin organization, and chromosome organization (Figure 3B; Supplementary Material Table S4). The enriched biological processes in the cell-type-specific proteomes confirmed that the quantified proteomes of PMCs and microspores accurately represent the biological processes aligned with the developmental stages of the isolated pollen cells.

### 3.2. The Heat-Induced Proteomics Changes in PMCs

From the PMC proteome comprising 3125 proteins, 60 HS-downregulated and 50 HS-upregulated DAPs were identified in ‘Black Vernissage’, while 41 HS-downregulated and 74 HS-upregulated DAPs were observed in ‘Micro-Tom’. We also compared the relative abundance of proteins involved in meiosis and heat shock proteins (HSPs) within the same varieties and between the two varieties under both non-heat-treated and heat-treated conditions (Table 1). Under non-heat-treated conditions, nearly all proteins showed no significant differences between the two varieties. However, due to the differential responses to HS, inter-varietal differences became significant under HS-treated conditions.

**Figure 4:**
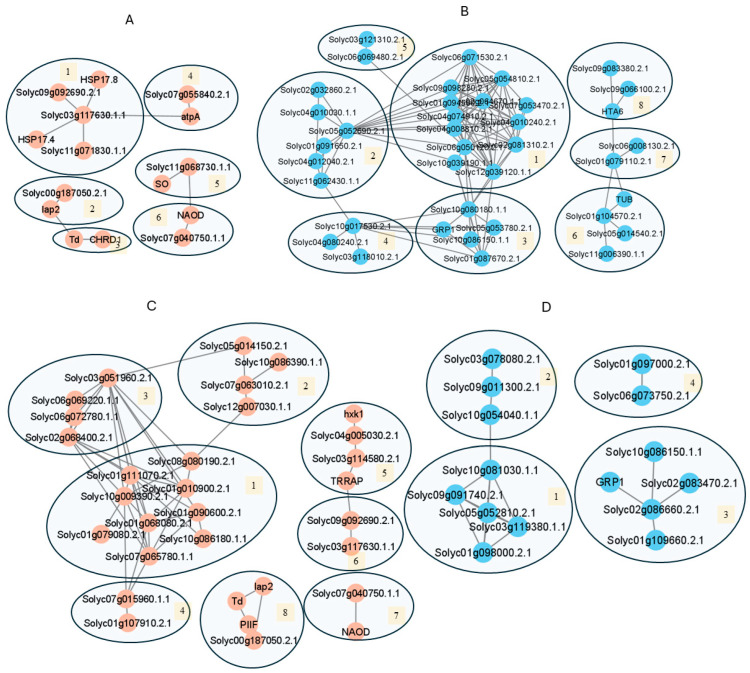
Protein association networks of HS-responsive differentially abundant proteins in pollen mother cells. The association networks were developed in STRING software (Version 11.5) using the database of *Solanum lycopersicum*. The medium confidence at 0.400 and full STRING network (the edges indicate both functional and physical protein associations) were selected. The network was analyzed using the Markov Cluster Algorithm (MCL) with an inflation parameter set to 3, and the resulting clusters were then visualized in Cytoscape. Data are provided in Supplementary Material Tables S5–S8. (**A**) HS-upregulated proteins in ‘Black Vernissage’. (**B**) HS-downregulated proteins in ‘Black Vernissage’. (**C**) HS-upregulated proteins in ‘Micro-Tom’. (**D**) HS-downregulated proteins in ‘Micro-Tom’.

The HS-downregulated and HS-upregulated proteins were searched in STRING to identify the protein interaction network in each variety (Figure 4A, Supplementary Materials Table S5). In ‘Black Vernissage’, the HS-upregulated proteins formed the largest cluster of heat shock proteins (HSPs), affecting protein folding (cluster 1) associated with ATP regeneration (cluster 4), aminopeptidases (cluster 2) associated with threonine dehydratase with the function of cellular detoxification (cluster 3), sulfite oxidase allowing the generation of ATP in oxidative phosphorylation in mitochondria (cluster 5) associated with DEAD-box helicases with a role in regulating programmed cell death, and protein translation (cluster 6).

The HS-downregulated proteins formed the largest network, mainly affecting protein translation/gene expression (Figure 4B, Supplementary Materials Table S6). The first interactome cluster comprises ribosomal proteins, eukaryotic translation initiation factors, ribonucleoprotein complex biogenesis, and ribosomal proteins (13 proteins in cluster 1), protein deneddylation in COP9 signalosome and the eIF-3 complex protein (Solyc05g052690.2.1) (cluster 2), U3 small nucleolar RNA-associated proteins involved in the processing of preribosomal RNA (cluster 3), RNA processing against stress conditions (Cluster 4), and mRNA transcriptional modification (cluster 5). The second interactive association network is composed of three clusters with proteins affecting DNA replication (cluster 6) and histone proteins (clusters 7, 8).

For MT, the HS-upregulated proteins formed a network of four clusters with proteins in the sporopollenin biosynthetic process (clusters 1–5), heat shock protein (cluster 6) and the DEAD box helicase involved in protein translation (cluster 7), and a cluster of metalloexopeptidases involved in glutathione metabolism (cluster 8) (Figure 4C, Supplementary Materials Table S7). The HS-downregulated proteins formed networks associated with protein translation/gene expression and RNA processing (cluster 1), and proteasomes (cluster 2), mRNA processing (cluster 3), and the glucan catabolic process (cluster 4) (Figure 4D, Supplementary Materials Table S8).

### 3.3. The Heat-Induced Proteomics Changes in Microspores

In the microspore proteomes, 2642 proteins were identified, including 59 HS-downregulated and 101 HS-upregulated DAPs in ‘Black Vernissage’ and 63 HS-downregulated and 97 HS-upregulated DAPs in ‘Micro-Tom’. These DAPs are involved in phenylpropanoid and pollen wall formation, flavonoid/isoflavonoid biosynthesis, amino acid metabolism, mitosis, proteolysis, and stress responses (Table 2). Most of these DAPs were detected within the same varieties, and both tomato varieties exhibited similar HS-induced changes. For many HS-induced DAPs identified in each individual variety, the relative protein abundance between the two varieties showed no significant differences under both non-heat-treated and HS-treated conditions. This contrasts with the PMCs, where most proteins exhibited varietal-specific HS-induced changes.

**Figure 5:**
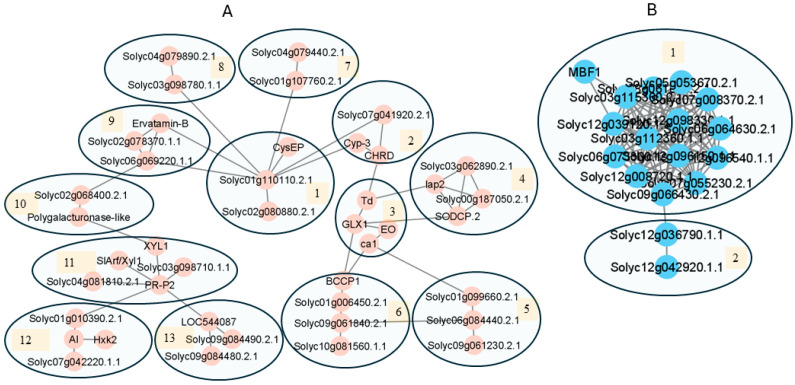

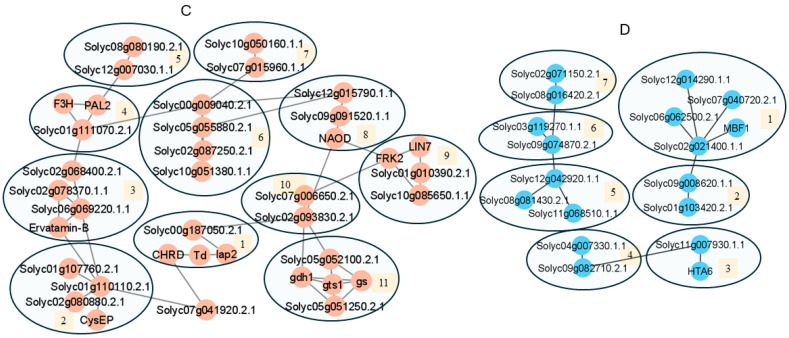
Protein association networks of HS-responsive differentially abundant proteins (DAPs) identified in tomato free microspore cells. The association network was developed in STRING software (Version 11.5) using the database of *Solanum lycopersicum*. The medium confidence at 0.400 and full STRING network (the edges indicate both functional and physical protein associations) were selected. The complex network was analyzed using the Markov Cluster Algorithm (MCL) with an inflation parameter set to 3, and the resulting clusters were then visualized in Cytoscape. Data are provided in Supplementary Material Tables S9–S12. (**A**) HS-upregulated proteins in ‘Black Vernissage’. (**B**) HS-downregulated proteins in ‘Black Vernissage’. (**C**) HS-upregulated proteins in ‘Micro-Tom’. (**D**) HS-downregulated proteins in ‘Micro-Tom’.

For each individual variety, the HS-downregulated and HS-upregulated DAPs were searched in STRING to identify the protein interactive network (Figure 5). In ‘Black Vernissage’, the HS-upregulated proteins formed a highly complex network with 13 interactome clusters. These clusters comprised proteins such as superoxide dismutase proteins (for the removal of reactive oxygen radicals); lactoylglutathione lyase (detoxification) and aminopeptidases (protein turnover) (clusters 1, 3, 4); proteolysis (clusters 2, 8, 7, 9, 11, 13); metabolic processes for cell wall (pollen exine) biosynthesis (clusters 10, 12); protein processing in the endoplasmic reticulum (HSPs) (cluster 5); and fatty acid biosynthesis and catabolism (cluster 6) (Figure 5A, Supplementary Material Table S9). The HS-downregulated DAPs formed the largest cluster in protein translation/gene expression, comprising ribosomes and ribonucleoprotein complex assembly (cluster 1). Cluster 2 is involved in oxidative phosphorylation (Figure 5B, Supplementary Material Table S10).

In Micro-Tom, the HS-upregulated proteins formed a network of 11 interconnected clusters (Figure 5C, Supplementary Material Table S11). These interactomes are involved in aminopeptidase activity (cluster 1), protease activity (clusters 2, 3), primary and secondary metabolic pathways (clusters 4, 5, 7, 9, 10), protein translation (clusters 6, 8) and glutamine metabolism (cluster 11). The HS-downregulated proteins formed clusters affecting the transcriptional regulation of stress responses (clusters 1, 2), chromosome organization (clusters 3, 4), mitochondrial respiratory chain complex, pollen inner wall development, and cell death (clusters 5, 6, 7) (Figure 5D, Supplementary Material Table S12).

### 3.4. The Cell- and Varietal-Specific HS-Induced Proteins

Proteins highly enriched in specific cells can serve as potential molecular markers for stress responses. We have identified a list of HS-responsive DAPs as candidate protein markers for pollen HS tolerance in tomatoes (Table 3). Twelve proteins showed consistent heat stress (HS)-induced changes in PMCs and microspores from both tomato varieties, indicating a shared HS-response mechanism. These proteins include acetylornithine deacetylase, involved in the cytosolic glutathione degradation pathway; glutathione (GSH), which is essential for redox regulation and detoxification of harmful compounds in plants [35]; epoxide hydrolases, which break down reactive epoxides to prevent cellular toxicity and DNA mutations; threonine ammonia lyase, which is involved in threonine biosynthesis; laccase, which plays a role in cell wall modification; thaumatin-like proteins (TLPs), which are associated with both biotic and abiotic stress responses [36]; and endoribonuclease, which acts on single-stranded mRNA and inhibits protein synthesis by cleaving mRNA. These proteins can be regarded as general HS-response markers influencing pollen development in tomato and potentially other plant species.

For genotype-specific HS protein markers, these proteins exhibited consistent changes in PMC and microspores in one of the two varieties. In ‘Black Vernissage’, two HS-upregulated DAPs were identified, including a methyl-binding domain (MBD) protein with a key role in regulating gene expression, chromatin organization, and epigenome maintenance, and a cathepsin B-like cysteine proteinase. In ‘Micro-Tom’, these DAPs are primarily involved in stress responses, including two chalcone synthase (CHS) enzymes, initiating the flavonoid biosynthetic pathway; the desiccation-related protein PCC13-62; glyoxal oxidase, which detoxifies reactive carbonyl species (RCS) and reduces reactive oxygen species (ROS) levels [37,38]; plant receptor-like kinases, acting as sentinels in plant defense responses [39]; and serine carboxypeptidase-like (SCPL) and subtilisin-like proteases, which contribute to plant defense and pollen tube growth.

For pollen developmental stage markers, these proteins exhibited HS-response changes in either PMCs or microspores from both tomato varieties. In PMCs, two HSPs were highly enriched under HS. In microspores, 25 HS-downregulated and 22 HS-upregulated proteins were specifically identified in both varieties. The HS-downregulated proteins include two transcription factors (Solyc02g062210.1.1, Solyc01g100750.2.1), proteases and stress response proteins (e.g., lactoylglutathione lyase, L-ascorbate peroxidase, major latex-like protein), enzymes associated with the primary metabolic pathways, including GDSL esterase/lipase and the secondary metabolites for stress tolerance (e.g., flavanone 3-hydroxylase, strictosidine synthase, Icc family phosphohydrolase, glutamine synthetase), and enzymes affecting cell wall modification (e.g., beta-galactosidase). The HS-downregulated proteins are primarily involved in gene expression, protein translation, proteases, cell wall cellulose remodeling, and kinases and phosphatases affecting protein post-translational modifications.

We also identified proteins showing significant HS-response changes only in one of the four cell samples. These are the proteins considered cell-type-specific and varietal-specific protein markers.

## 4. Discussion

Pollen thermotolerance is a critical trait in tomatoes, significantly influencing fruit set, yield, and quality. In this study, two tomato varieties were subjected to HS over a three-week period, covering the key stages of pollen development. The ‘Black Vernissage’ variety demonstrated greater tolerance than ‘Micro-Tom’, as evidenced by less severe reductions in pollen productivity under HS. This aligns with our observations, indicating that ‘Black Vernissage’ is more productive than ‘Micro-Tom’ and many other tomato varieties under both mildly and highly heat-treated conditions (unpublished data, Zhou et al., Tennessee State University).

In this study, we identified HS-induced proteomes in PMCs and microspores. PMCs were collected during meiosis I and II but before the tetrad stage, and proteomics analysis confirmed the differential expression of meiosis-specific proteins between the two tomato varieties. Notably, the meiosis-specific ASY3 (Solyc01g079080) and the tomato MEI2-like RNA (TML)-binding protein (Solyc01g110240.1.1) only showed HS-induced increases in the ‘Micro-Tom’ variety. MEI2 is a transcription factor that regulates the expression of genes essential for meiosis initiation and progression while preventing the degradation of meiosis-specific mRNAs. Initially identified in fission yeast (*Schizosaccharomyces pombe*), subsequent studies have confirmed that plant homologs of MEI2 play a critical role as master regulators of meiosis in plants [40,41]. Conversely, two other meiosis-specific proteins, including the COP9 signalosome (Solyc11g062430.1.1, −1.15, *p* < 0.0001 in MT), were repressed in ‘Micro-Tom’ under HS. The COP9 signalosome, which regulates ubiquitin-mediated degradation, also influences synaptonemal complex (SC) assembly between paired chromosomes, impacting chromosome synapsis [42,43,44]. All eight subunits of the COP9 signalosome complex were quantified in the PMCs, but none of these proteins exhibited significant changes from the non-heat-treated to heat-treated conditions in ‘Black Vernissage’.

In ‘Black Vernissage’, the kinetochore protein NDC80 was decreased (Solyc01g104570.2.1, −0.92-fold, *p* = 0.04), while it remained unaffected in ‘Micro-Tom’ under HS. This protein plays a crucial role in organizing and stabilizing microtubule–kinetochore interactions at centromeres and is essential for proper chromosome segregation during meiosis. Meiotic centromeres have been found to be highly sensitive to HS [45,46]. In *Arabidopsis thaliana*, the elevated temperatures were shown to reduce the levels of centromeric histone and the kinetochore protein BMF1 at meiotic centromeres, leading to a prolonged duration of meiotic divisions [47,48]. Crhak et al. [47] suggested that centromeres and kinetochores may represent critical bottlenecks in plant adaptation to increasing temperatures. In conclusion, these meiosis-specific proteins, with their distinct responses to HS depending on the variety, may serve as key targets for improving HS tolerance in sensitive tomato varieties.

In this study, microspores were harvested at the unicellular pre-mitotic stage. HS induced significant changes in proteins associated with pollen wall formation, which is essential for protecting male gametophytes and ensuring plant reproduction [4,49]. The pollen wall primarily consists of three layers: the pollen coat, the outer exine layer, and the inner intine layer. In both tomato varieties, HS significantly increased hydroxycinnamoyl-CoA shikimate/quinate hydroxycinnamoyltransferase (HCT) (Solyc07g015960.1.1: 2.46-fold, *p* = 0.03 in ‘Black Vernissage’ and 3.44-fold, *p* < 0.0001 in ‘Micro-Tom’). This enzyme plays a key role in lignin biosynthesis, and its alteration can affect lignin composition, thereby impacting pollen exine formation [50]. Two enzymes involved in xylan modification were significantly affected by HS in ‘Micro-Tom’ but not in ‘Black Vernissage’: xyloglucan endotransglucosylase/hydrolase 9 (XTH) (Solyc03g093110.2.1: 1.38-fold, *p* = 0.04 in ‘Micro-Tom’) and acetyl xylan esterase A (Solyc01g102330.2.1: −1.92-fold, *p* < 0.0001 in ‘Micro-Tom’). Xylan composition influences microspore cell wall formation and pollen exine patterning, which are critical for proper pollen grain maturation. The primary component of the intine layer is pectin, which plays a crucial role in the polar growth of pollen tubes [51,52,53]. Exogenous pectin methylesterase (PME) application has been shown to thicken the apical cell wall and inhibit pollen tube growth. PMEs function in conjunction with PME inhibitors (PMEIs), which regulate cell wall stability. In this study, the pectinesterase (Solyc07g017600.2.1: 0.96-fold, *p* = 0.13 in BV; 1.65-fold, *p* < 0.0001 in ‘Micro-Tom’) and pectinesterase inhibitor (Solyc01g097660.2.1: 4.03-fold, *p* < 0.0001 in ‘Black Vernissage’; 4.19-fold, *p* < 0.0001 in ‘Micro-Tom’) were induced by HS in both varieties. These observed increases in these enzymes may affect the intine structure of mature pollen grains. Together, these cell wall-associated proteins influence pollen wall formation, ultimately affecting pollen maturation, viability, and germination.

Besides the cell-specific biological processes, HS induced a significant reduction in protein translation, which is one of the most energetically expensive processes a cell undertakes. One of the heat stress tolerance mechanisms is that plants exhibit a strong inhibition of general translation, while selectively promoting the translation of stress-responsive proteins essential for cell survival and recovery [54,55]. Both PMCs and microspores from the two tomato varieties exhibited decreases in ribosomal proteins, translation initiation factors, and other proteins involved in translational machinery. Overall, these changes will lead to a decrease in translation efficiency. These results are consistent with our previous proteomics analysis of the PMCs in the HS-tolerant ‘Maxifort’ tomato variety [22].

Heat stress induces the accumulation of misfolded proteins and damaged organelles in plant cells, impairing plant growth and development [22,28,56]. Increased activity of the proteolytic system (i.e., the ubiquitin–proteasome system and autophagy–lysosomal system) and repair system (i.e., molecular chaperones) promotes the maintenance of protein homeostasis [57,58,59]. Plants possess a complex network of proteases and protease inhibitors (PIs) that regulate protein degradation and activation [60,61]. In meiotic PMCs, 22 DAPs belonging to the protease group were identified, including two Kunitz-type protease inhibitors (Solyc03g098790.1.1 and Solyc03g098710.1.1) that were upregulated under HS in both varieties. However, the remaining 20 proteins showed inconsistent changes. In the microspores, 33 DAPs were identified with consistent HS-induced changes, though only 11 proteins reached significant levels in both varieties. The ‘Black Vernissage’ variety exhibited a larger number of HS-induced DAPs (19 proteins) compared to ‘Micro-Tom’ (12 proteins), while there were fewer HS-repressed proteins in ‘Black Vernissage’ (2 proteins) than in ‘Micro-Tom’ (5 proteins). These findings highlight the robust proteolytic activity under HS and the greater genotypic differences in PMCs.

In the microspores, HSBP1 (Solyc12g099570.1.1) was identified but did not meet the qualification criteria. Although heat shock factors (HSFs) were not identified, a large number of HSPs increased in HS-treated PMCs and microspores. Furthermore, the abundance of three HSPs (Solyc06g076520.1.1, Solyc06g076570.1.1, Solyc01g099660.2.1) varied between the two varieties, with ‘Black Vernissage’ generally containing higher levels of these proteins under HS. These results indicate that these HSPs contributed to the enhancement of thermotolerance at a greater magnitude in ‘Black Vernissage’ compared to ‘Micro-Tom’.

Additionally, ascorbate peroxidase and catalase can form a regulatory loop mechanism to control oxidative species under HS [62,63,64]. In ‘Black Vernissage’, three antioxidant enzymes were HS-induced, with two superoxide dismutase isoforms (Solyc11g066390.1.1, Solyc03g062890.2.1) reaching significant levels. Ascorbate peroxidase (Solyc04g074640.2.1) was induced in both ‘Black Vernissage’ and ‘Micro-Tom’ but reached a significant level only in ‘Micro-Tom’. Other proteins, including thaumatin-like protein (Solyc04g079890.2.1), defensin protein (Solyc07g007730.2.1), and late embryogenesis-abundant protein (Solyc07g053360.2.1), were consistently induced by HS in both varieties in PMCs and more so in microspores, at a more pronounced level in ‘Black Vernissage’. These findings suggest a greater capacity for thermotolerance in ‘Black Vernissage’ compared to ‘Micro-Tom’.

In this study, we effectively addressed the challenges of quantitative proteomics analysis using small amounts of proteins and identified spatially and temporarily resolved cell-type proteomics in anther tissues. However, the results are based on the quantitative changes (relative abundance) in proteins measured by tryptic peptides in a bottom-up proteomics approach. Proteoforms, including post-translational modifications, also play a significant role in regulating biological activities, and in some cases, this role may be even greater [65,66]. In this study, the identified HS-induced DAPs, such as protein kinases and phosphatases, proteasome, and those involved in protein deneddylation, all contributed to facilitating post-translational modifications during pollen development against elevated temperature stress. To provide a more comprehensive understanding of the proteomic changes related to pollen heat tolerance, future work will incorporate top-down proteomics technology and targeted proteomics analysis of the DAPs identified in this study.

## 5. Conclusions

In this study, we identified the proteomes of PMCs and microspores using pure cell populations isolated via LCM. The heat stress-induced changes in protein abundance levels offered new insights into the extensive proteome reprogramming that occurs during pollen development under heat stress. Specifically, proteins involved in ensuring the fidelity of the meiotic cell cycle and stress-related proteins were more abundant in ‘Black Vernissage’ than in ‘Micro-Tom’. The meiotic PMCs of ‘Black Vernissage’ were enriched with a greater variety and higher abundance levels of HSPs. By the free microspore stage, many of the inter-varietal differences in protein expression diminished, with a greater number of proteins being either induced or repressed in both varieties. These developmental stage-specific findings highlight the importance of single-cell-type proteomics analysis in identifying heat stress tolerance proteins. Overall, heat stress induced the repression of protein translation while amplifying the selective expression of genes crucial for acquiring stress tolerance. The dynamic proteomic changes in the meiotic pollen mother cells, along with the inter-varietal differences, highlight that future research on male heat tolerance should place emphasis on the early stages. The HS-induced proteins and the encoding genes identified in this study can be used to develop molecular markers for genotyping heat tolerance, and these genes will be selected as targets of gene (genome) editing to develop tolerant genotypes.

## Figures and Tables

**Table 1:** Heat-induced proteins involved in meiosis and heat responses in pollen mother cells from ‘Black Vernissage’ and ‘Micro-Tom’.

Accession	BV ^1^ (HT/Ctr) ^3^	MT ^2^ (HT/Ctr)	BV/MT (Ctr)	BV/MT (HT)	Protein Name
Meiosis					
Solyc06g069480	−0.8 *	0.3	0.3	−0.8 *	DExH-box ATP-dependent RNA helicase DExH14
Solyc01g091650	−0.8 *	−0.1	0.0	−0.7	COP9 signalosome complex subunit 1
Solyc01g005820	−0.6	0.0	−0.1	−0.7 *	Splicing factor arginine/serine-rich 4
Solyc09g072570	−0.5 *	0.2	0.0	−0.7 *	Pre-mRNA-splicing factor SLU7-A
Solyc05g056230	0.6 *	−0.1	−0.3	0.5 *	Calreticulin 2 calcium-binding protein
Solyc01g104570	−0.9 *	0.5	0.4	−1.0 *	Kinetochore protein ndc80
Solyc06g069480	−0.8 *	0.3	0.3	−0.8 *	ATP-dependent RNA helicase
Solyc10g080740	−0.8	0.0	0.0	−0.8 *	Smg-7 homolog nonsense-mediated mRNA decay factor
Solyc01g091650	−0.8 *	−0.1	0.0	−0.7	COP9 signalosome complex subunit 1
Solyc11g070140	−0.8 *	0.1	0.0	−0.8 *	Cell division protein kinase 2
Solyc02g084700	−0.5 *	0.0	0.2	−0.3	MORC family CW-type zinc finger 3
Solyc07g064970	−0.5 *	0.4	0.3	−0.5 *	Microtubule-associated protein MAP65-1a
Solyc11g065180	−0.5 *	0.1	0.2	−0.4	THUMP domain-containing protein
Solyc01g079080	−0.4	0.7 *	0.1	−1.1 *	Meiosis-specific protein ASY3
Solyc07g018300	−0.3	0.0	−0.1	−0.5 *	Single-stranded DNA-binding protein p30 subunit
Solyc08g075840	−0.3	0.5 *	0.4	−0.3	Single-stranded DNA-binding replication protein A large subunit
Solyc07g045010	0.0	−0.5 *	−0.6 *	−0.2	Protein JASON
Solyc04g007120	0.5 *	0.2	−0.1	0.2	UV excision repair protein RAD23
Heat shock proteins					
Solyc04g009770	−0.8 *	−0.1	0.1	−0.5	DNAJ chaperone
Solyc05g055160	−0.7 *	0.1	0.2	−0.6 *	DNAJ chaperone
Solyc11g006460	−0.6 *	−0.2	0.1	−0.3	DNAJ chaperone
Solyc03g121640	0.2	0.1	0.4	0.5 *	chaperonin
Solyc03g116790	0.3	0.4	−0.3	−0.5 *	Chaperone protein dnaJ
Solyc10g084300	0.3	0.6 *	0.0	−0.3	Chaperone protein dnaJ 3
Solyc09g007630	0.4	0.1	0.2	0.5 *	Chaperone DnaJ
Solyc03g117630	1.5 *	0.8	−0.4	0.3	Heat shock protein
Solyc08g082820	0.5 *	0.1	−0.2	0.2	Heat shock protein
Solyc01g109890	0.5 *	0.4	0.2	0.3	Chaperone protein dnaJ 10
Solyc09g005120	0.6 *	0.2	−0.1	0.3	Chaperone protein DnaJ
Solyc11g020040	0.6 *	0.4	0.1	0.3	Chaperone DnaK
Solyc03g082920	0.7 *	0.2	−0.2	0.3	Heat shock protein
Solyc02g088610	0.7 *	0.4	−0.2	0.1	ATP-dependent chaperone ClpB
Solyc04g081570	0.8 *	0.3	0.1	0.6 *	Chaperone protein htpG
Solyc11g071830	1.0 *	0.0	−0.1	0.8 *	Chaperone protein dnaj
Solyc08g062340	1.2 *	0.2	−0.2	0.8 *	Class II small heat shock protein Le-HSP17.6
Solyc06g076520	1.2 *	−0.2	0.4	1.8 *	Class I heat shock protein
Solyc03g117630	1.5 *	0.8 *	−0.4	0.3	Heat shock protein
Solyc03g123540	1.7 *	0.8 *	0.9	1.8 *	Class II heat shock protein
Solyc04g014480	2.7 *	1.1 *	−0.1	1.5 *	Class I heat shock protein 3

* Differentially abundant proteins (DAPs) defined by a fold change greater than two standard deviations (SDs) and using an FDR-adjusted *p*-value threshold of ≤0.05. When comparing the two tomato varieties, positive values indicate proteins with higher abundance in ‘Black Vernissage’ than in ‘Micro-Tom’, whereas the negative values show lower abundance in ‘Black Vernissage’ than in ‘Micro-Tom’. ^1,2^ Tomato varieties, BV for ‘Black Vernissage’, MT for ‘Micro-Tom’. ^3^ HT—heat-treated condition; Ctr—non-heat-treated control condition.

**Table 2:** Heat-induced proteins and the biological process in microspores from ‘Black Vernissage’ and ‘Micro-Tom’.

Accession	BV ^1^ (HT/Ctr) ^3^	MT ^2^ (HT/Ctr)	BV/MT (Ctr)	BV/MT (HT)	Protein Names
Phenylpropanoids and pollen wall formation					
Solyc10g050160	2.29 *	3.02 *	−0.07	−0.93	Caffeoyl-CoA 3-O-methyltransferase
Solyc07g015960	2.46 *	3.44 *	0.30	−0.81	Hydroxycinnamoyl CoA quinate transferase
Solyc12g096770	−2.06 *	−0.94	0.43	−0.82	Hydroxycinnamoyl CoA quinate transferase
Solyc04g081520	−1.71 *	−0.87	1.31 *	0.34	Laccase-16
Solyc12g094460	1.30	2.43 *	−0.02	−1.28	Laccase-2
Solyc05g056170	1.45	2.13 *	0.66	−0.15	Phenylalanine ammonia-lyase
Solyc01g112000	2.05 *	3.03 *	0.62	−0.49	Expansin-like protein
Solyc12g015690	2.40 *	1.90 *	0.20	0.57	Fasciclin-like arabinogalactan protein 10
Solyc11g005490	−1.83 *	−2.26 *	−0.57	−0.27	Fasciclin-like arabinogalactan protein 18
Solyc06g073750	−2.26 *	−2.84 *	−0.45	0.00	Beta-D-glucosidase
Solyc11g072230	−2.71 *	−1.60	−0.03	−1.27	Glucan endo-1 3-beta-glucosidase 3
Solyc06g076170	−2.25 *	−3.06 *	−0.99 *	−0.30	Glucan endo-1 3-beta-glucosidase
Solyc11g044910	1.68 *	1.37	0.23	0.41	Beta-xylosidase 1
Solyc10g047030	1.63 *	0.89	0.90 *	1.52 *	Beta-xylosidase 4
Solyc01g102330	−0.26	−1.92 *	−0.27	1.27	Acetyl xylan esterase A
Solyc01g087280	1.78 *	0.92	−0.88	−0.15	Polygalacturonase A
Solyc01g097660	4.03 *	4.19 *	−0.02	−0.31	Pectinesterase inhibitor
Solyc02g093580	−1.13	−1.80 *	0.29	0.83	Pectate lyase
Flavonoid/isoflavonoid biosynthesis					
Solyc02g083860	1.98 *	3.24 *	1.18 *	−0.21	Flavanone 3-hydroxylase
Solyc01g111070	1.20	3.25 *	1.14 *	−1.03	Chalcone synthase family protein
Fatty acid metabolism					
Solyc09g061840	2.17 *	2.02 *	0.38	0.40	3-ketoacyl CoA thiolase 1
Solyc05g056290	1.72 *	−0.11	−1.23	0.47	Acetyl-CoA carboxylase
Solyc01g006450	1.76 *	1.34	0.01	0.31	Enoyl reductase
Solyc12g100120	1.47	2.00 *	0.05	−0.61	Acetyl-CoA carboxylase-like protein
Solyc09g092450	1.16	2.46 *	0.51	−0.91	Long-chain fatty acid CoA ligase
Amino acid metabolism					
Solyc08g076970	1.82 *	2.34 *	0.36	−0.29	Acetylornithine deacetylase
Solyc09g008670	1.69 *	2.91 *	0.45	−0.89	Threonine ammonia-lyase
Solyc06g064550	−1.75 *	−1.37	−0.55	−1.06	Aspartokinase–homoserine dehydrogenase
Solyc10g078550	2.50 *	2.52 *	0.38	0.23	Glutamate dehydrogenase
Solyc05g052100	0.35	1.92 *	0.33	−1.37 *	Glutamate dehydrogenase
Solyc05g051250	2.27	1.81 *	−0.21	0.12	Glutamine synthetase
Solyc01g080280	1.23	2.25 *	0.44	−0.71	Glutamine synthetase
Solyc04g014510	1.52	2.63 *	0.67	−0.56	Glutamine synthetase
Protease activity					
Solyc00g187050	1.75 *	1.99 *	0.43	0.06	Leucyl aminopeptidase
Solyc12g010020	2.11 *	2.18 *	0.32	0.13	Leucyl aminopeptidase
Solyc02g080880	1.76 *	2.13 *	−0.12	−0.62	Aspartic proteinase
Solyc06g069220	4.60 *	5.98 *	1.61	0.10	Aspartic proteinase nepenthesin I
Solyc01g107760	2.98 *	2.73 *	−0.18	−0.06	Cathepsin B-like cysteine proteinase
Solyc03g111730	2.41 *	4.10 *	0.22	−1.59	Cathepsin B-like cysteine proteinase
Solyc07g053460	3.73 *	4.38 *	0.67	−0.12	Cathepsin B-like cysteine proteinase
Solyc07g041920	3.68 *	3.16 *	0.09	0.48	Cathepsin L-like cysteine proteinase
Solyc01g110110	3.16 *	5.28 *	1.38 *	−0.86	Cysteine proteinase cathepsin F
Solyc11g008240	3.27 *	2.74 *	−0.70	−0.29	SKP1-like protein
Solyc09g084490	3.38 *	3.32 *	1.80	1.73 *	Proteinase inhibitor I
Solyc03g098680	3.30 *	4.51 *	0.71	−0.64	Kunitz trypsin inhibitor
Solyc04g081810	3.40 *	1.92 *	0.99	2.35 *	Subtilisin-like protease
Solyc02g092670	2.81 *	2.91 *	0.54	0.31	Subtilisin-like protease
Solyc07g041900	1.85 *	1.19	−0.40	0.13	Cathepsin L-like cysteine proteinase
Solyc03g098780	1.89 *	0.06	−0.06	1.64 *	Kunitz-type protease inhibitor
Solyc03g098790	2.26 *	−0.04	0.16	2.34 *	Kunitz-type protease inhibitor
Solyc03g098760	2.83 *	0.79	0.91	2.81 *	Kunitz-type protease inhibitor-like protein
Solyc03g098710	2.36 *	1.25	1.24 *	2.22 *	Kunitz-type proteinase inhibitor A4
Solyc02g091410	1.63 *	0.72	−0.68	0.09	Pro-apoptotic serine protease nma111-like protein
Solyc09g084480	2.26 *	1.59	1.49 *	2.02 *	Proteinase inhibitor I
Solyc03g020030	2.34 *	1.08	0.54	1.68 *	Proteinase inhibitor II
Solyc04g079440	1.62 *	0.73	0.16	0.93	Serpin
Solyc00g071180	3.89 *	1.14	2.16	4.78 *	Cysteine proteinase inhibitor
Solyc06g072570	−2.16 *	−0.54	−0.44	−2.18 *	Ubiquitin-conjugating enzyme E2 1
Solyc01g096450	−1.03	−1.87 *	−0.42	0.29	Aspartic proteinase nepenthesin-1
Solyc11g020960	−1.02	−2.45 *	−0.14	1.16	Proteinase inhibitor II
Solyc01g111400	−1.43	−1.96 *	−0.44	−0.04	Subtilisin-like protease
Solyc11g065210	−0.91	−2.43 *	0.17	1.56	Ubiquitin carboxyl-terminal hydrolase
Solyc06g074850	0.02	2.11 *	0.53	−1.69	Serine carboxypeptidase
Solyc08g079910	1.27	2.89 *	0.27	−1.47	Subtilisin-like protease
Transcription factors					
Solyc04g071620	3.47 *	0.65	−1.47	1.22	ASR4
Solyc02g062210	1.98 *	1.46	1.57 *	1.96 *	NAC transcription factor
Solyc01g100750	2.05 *	1.75	0.21	0.38	Susceptibility homeodomain transcription factor
Mitosis					
Solyc02g062410	3.04 *	3.94 *	0.72	−0.31	Nuclear movement protein nudc
Solyc06g084440	1.88 *	1.66	0.08	0.17	Nuclear protein localization 4
Solyc04g007330	−1.84 *	−2.35 *	0.56	0.94	HORMA domain-containing protein expressed
Solyc06g083870	−1.78 *	−1.49	0.29	−0.13	Structural maintenance of the chromosome 1 protein
Solyc08g078320	−1.01	−1.82 *	−0.01	0.67	E3 ubiquitin–protein ligase HOS1
Solyc02g078290	−1.98 *	−2.67 *	0.26	0.82	Myosin-like protein
Responses to stress					
Solyc06g076520	−1.47	−2.35 *	0.64	1.39	Class I heat shock protein
Solyc06g076570	−1.18	−1.82 *	0.53	1.05	Class I heat shock protein
Solyc01g099660	2.08 *	0.84	0.01	1.11	Heat shock protein
Solyc03g111860	1.02	2.25 *	0.50	−0.86	Glyoxal oxidase
Solyc11g069040	1.61 *	1.26	−0.22	0.00	Lactoylglutathione lyase
Solyc04g074640	1.24	2.16 *	0.80	−0.25	L-ascorbate peroxidase
Solyc11g066390	1.63 *	0.58	0.10	1.02	Superoxide dismutase
Solyc03g062890	1.84 *	1.25	0.38	0.83	Superoxide dismutase
Solyc04g079890	3.69 *	4.23 *	0.99	0.33	Thaumatin-like protein
Solyc07g007730	2.65 *	3.00 *	0.86	0.38	Defensin protein
Solyc07g053360	2.69 *	2.39 *	0.96	1.13	Late embryogenesis-abundant protein

* Heat stress induced differentially abundant proteins (DAPs), which were defined by a fold change greater than two standard deviations (SDs) and using an FDR-adjusted *p*-value threshold of ≤0.05. When comparing the two tomato varieties, positive values indicate proteins with higher abundance in ‘Black Vernissage’ than in ‘Micro-Tom’, whereas negative values show lower abundance in ‘Black Vernissage’ than in ‘Micro-Tom’. ^1,2^ Tomato varieties, BV for ‘Black Vernissage’, MT for ‘Micro-Tom’. ^3^ HT—heat-treated condition; Ctr—non-heat-treated control condition.

**Table 3:** The heat stress-induced proteins in pollen mother cells and microspores in ‘Black Vernissage’ and ‘Micro-Tom’.

Accession	BV ^1^ Microspore	BV PMC ^3^	MT ^2^ Microspore	MT PMC	Description
Variety specific					
Solyc04g080880.2.1	1.37 *	0.82 *	0.60	−0.19	Cathepsin B-like cysteine proteinase
Solyc06g074850.2.1	0.02	0.41	2.11 *	1.03 *	Serine carboxypeptidase
Solyc06g068140.2.1	1.18 *	0.97 *	0.51	−0.17	Methyl-binding domain protein
Solyc05g013720.2.1	1.38	0.26	2.26 *	1.24	Alpha-galactosidase
Solyc01g090600.2.1	1.01	0.43	1.55 *	1.41 *	Chalcone synthase 3 protein
Solyc01g111070.2.1	1.20	−0.15	3.25 *	0.77 *	Chalcone synthase
Solyc01g010900.2.1	0.86	0.60	1.19 *	1.10 *	Cytochrome P450
Solyc01g107910.2.1	0.24	0.23	1.64 *	1.23 *	Caffeoyl CoA 3-O-methyltransferase
Solyc01g099580.1.1	0.96	0.10	4.08 *	2.41 *	Desiccation-related protein PCC13-62
Solyc07g006660.1.1	1.19	0.50	0.96 *	1.21 *	Glyoxal oxidase
Solyc05g050700.1.1	0.55	0.40	1.09 *	0.73 *	LRR receptor-like serine/threonine–protein kinase, RLP
Solyc03g044150.2.1	−0.66	0.37	−1.17 *	−0.72 *	Subtilisin-like protease
Pollen developmental stage-specific					
Solyc04g014480.2.1	0.16	2.68 *	−0.27	1.06 *	Class I heat shock protein 3
Solyc03g123540.2.1	−0.22	1.69 *	0.00	0.77 *	Class II heat shock protein
Solyc03g111730.2.1	2.41 *	0.21	4.10 *	0.50	Cathepsin B-like cysteine proteinase
Solyc04g081810.2.1	3.40 *	0.44	1.92 *	0.40	Subtilisin-like protease
Solyc02g082300.2.1	1.38 *	0.42	1.97 *	0.55	AT-hook motif nuclear-localized protein 2
Solyc02g062210.1.1	1.98 *	−0.04	1.46 *	−0.01	NAC transcription factor
Solyc06g084440.2.1	1.88 *	−0.05	1.66 *	0.20	Nuclear protein localization 4
Solyc01g100750.2.1	2.05 *	−0.08	1.75 *	−0.30	Susceptibility homeodomain transcription factor
Solyc09g061230.2.1	2.07 *	0.14	1.61 *	0.06	Cytochrome b5
Solyc02g083860.2.1	1.98 *	−0.16	3.24 *	0.61	Flavanone 3-hydroxylase
Solyc01g058390.2.1	1.33 *	−0.06	2.37 *	0.10	Galactokinase
Solyc05g012540.2.1	1.54 *	0.23	2.22 *	0.02	Heparanase
Solyc11g066290.1.1	2.24 *	0.40	2.18 *	0.35	Icc family phosphohydrolase
Solyc05g051850.2.1	2.31 *	0.07	1.18 *	−0.03	Inositol-3-phosphate synthase
Solyc01g110060.2.1	2.34 *	0.01	2.52 *	−0.37	Purple acid phosphatase
Solyc00g058900.1.1	1.68 *	0.64	3.56 *	0.35	GDSL esterase/lipase
Solyc11g071800.1.1	0.84 *	0.55	0.97 *	0.57	Strictosidine synthase family protein
Solyc07g042220.1.1	2.62 *	−0.29	3.51 *	0.18	Beta-galactosidase
Solyc05g054590.2.1	1.73 *	0.29	1.64 *	−0.10	Bifunctional polymyxin resistance protein ArnA
Solyc05g051250.2.1	2.27 *	−0.05	1.81 *	−0.36	Glutamine synthetase
Solyc11g069040.2.1	1.61 *	0.35	1.26 *	0.26	Lactoylglutathione lyase
Solyc04g074640.2.1	1.24 *	0.52	2.16 *	0.21	L-ascorbate peroxidase
Solyc11g073210.1.1	1.39 *	−0.47	3.37 *	0.09	Legumin 11S-globulin
Solyc07g008710.2.1	2.79 *	−0.40	2.10 *	0.23	Major latex-like protein
Solyc01g068040.1.1	−2.36 *	0.14	−1.70 *	−0.35	3-5 exonuclease/nucleic acid binding protein
Solyc03g115360.2.1	−1.80 *	−0.44	−1.55 *	−0.30	40S ribosomal protein S19-like
Solyc02g021400.1.1	−1.25 *	−0.58	−1.92 *	−0.45	40S ribosomal protein S28
Solyc12g096150.1.1	−1.66 *	−0.23	−0.96 *	−0.28	60S ribosomal protein L13
Solyc09g066430.2.1	−1.77 *	−0.42	−0.91 *	−0.27	60S ribosomal protein L14
Solyc12g008720.1.1	−1.69 *	0.00	−1.08 *	−0.46	60S ribosomal protein L31
Solyc04g007330.1.1	−1.84 *	0.17	−2.35 *	0.01	DNA-binding HORMA
Solyc09g008620.1.1	−1.57 *	−0.34	−1.91 *	0.10	Polyadenylate-binding protein
Solyc08g061320.2.1	−1.90 *	0.36	−2.26 *	−0.41	Smr domain-containing protein
Solyc06g083870.2.1	−1.78 *	−0.09	−1.49 *	0.07	Structural maintenance of the chromosome 1 protein
Solyc01g103420.2.1	−1.08 *	−0.23	−1.96 *	0.18	Upf3 regulator of nonsense transcripts-like protein B
Solyc06g064480.2.1	−2.06 *	0.01	−2.04 *	0.40	Protein TAP1
Solyc06g051010.1.1	−1.51 *	0.35	−2.08 *	−0.21	Asparagine amidase A
Solyc01g111400.2.1	−1.43 *	0.08	−1.96 *	−0.17	Subtilisin-like protease
Solyc01g096450.2.1	−1.03 *	0.59	−1.87 *	−0.58	Aspartic proteinase nepenthesin-1
Solyc08g078320.2.1	−1.01 *	−0.66	−1.82 *	0.16	E3 ubiquitin–protein ligase HOS1
Solyc07g045010.2.1	−1.93 *	0.02	−1.98 *	−0.46	Dentin sialophosphoprotein-like
Solyc11g066130.1.1	−1.82 *	0.60	−2.19 *	−0.54	Thaumatin-like protein
Solyc01g111280.2.1	−1.17 *	−0.14	−2.17 *	−0.37	Cold shock protein-1
Solyc11g072230.1.1	−2.71 *	0.46	−1.60 *	−0.11	Glucan endo-1 3-beta-glucosidase 3
Solyc06g076170.2.1	−2.25 *	−0.39	−3.06 *	−0.20	Glucan endo-1 3-beta-glucosidase
Solyc12g096770.1.1	−2.06 *	0.09	−0.94 *	0.19	Hydroxycinnamoyl CoA quinate transferase
Solyc11g005490.1.1	−1.83 *	0.05	−2.26 *	−0.50	Fasciclin-like arabinogalactan protein
Solyc06g064550.2.1	−1.75 *	0.04	−1.37 *	0.25	Aspartokinase
Solyc07g040720.2.1	−1.55 *	0.01	−2.08 *	−0.12	Serine/threonine kinase
Solyc10g051090.1.1	−2.37 *	0.39	−2.82 *	−0.04	Serine/threonine–protein phosphatase
Variety- and cell-type-specific					
Solyc06g074720.2.1	2.15 *	−0.44	0.90	−0.24	FHA domain-interacting nucleolar phosphoprotein
Solyc09g005910.2.1	1.87 *	−0.12	0.66	0.47	Mg^2+^-dependent phosphatidate
Solyc10g083650.1.1	0.89 *	0.13	0.05	−0.79 *	Peroxiredoxin ahpC/TSA family
Solyc04g082010.1.1	2.11 *	0.19	0.62	−0.23	Plastocyanin
Solyc11g066390.1.1	1.63 *	0.22	0.58	−0.03	Superoxide dismutase
Solyc08g005060.2.1	−0.12	0.89 *	−0.32	−0.57	SnRK1-interacting protein 1
Solyc06g034120.2.1	−0.96	0.92 *	−0.01	−0.36	Alcohol dehydrogenase
Solyc11g069180.1.1	0.55	0.86 *	0.80	−0.11	Isovaleryl-CoA dehydrogenase
Solyc01g111660.2.1	−0.30	0.94 *	0.02	−0.38	Aquaporin-like protein
Solyc08g075870.2.1	−0.04	0.84 *	−0.03	0.67	Dehydration-responsive family protein-like
Solyc01g081270.2.1	0.62	0.87 *	−0.17	0.35	Glutathione S-transferase
Solyc00g009040.2.1	1.50	−0.33	2.40 *	0.41	Polyadenylate-binding protein 2
Solyc01g110210.1.1	−0.11	−0.22	1.11 *	0.98	Terminal ear1-like 2 protein
Solyc06g073190.2.1	0.91	−0.10	2.82 *	0.12	Fructokinase-like
Solyc11g073200.1.1	1.59	0.11	3.55 *	0.02	Legumin 11S-globulin
Solyc10g085650.1.1	0.77	0.25	1.89 *	0.16	Beta-fructofuranosidase insoluble isoenzyme 2
Solyc02g062460.2.1	0.58	0.06	1.98 *	0.12	2-oxoglutarate-dependent dioxygenase
Solyc09g065840.2.1	−0.53	−0.24	−0.11	0.73 *	Single-stranded DNA-binding protein
Solyc10g009390.2.1	0.77	0.14	0.13	0.89 *	Cytochrome P450
Solyc01g088430.2.1	0.26	0.17	0.11	0.71 *	Fatty acid hydroxylase
Solyc04g005030.2.1	−0.68	0.72	−0.03	0.79 *	Phosphoglucomutase
Solyc10g078490.1.1	0.20	0.40	−0.15	0.86 *	Aquaporin
Solyc09g075120.2.1	0.85	−0.13	0.47	0.70 *	Bifunctional polymyxin resistance protein ArnA
Solyc05g015390.2.1	−0.26	0.58	−0.65	0.81 *	REF-like stress-related protein 1/Rubber elongation factor
Solyc12g096540.1.1	−1.66 *	−0.52	0.08	−0.58	40S ribosomal protein S10-like
Solyc07g055230.2.1	−2.01 *	−0.15	−0.46	−0.31	50S ribosomal protein L5
Solyc05g053670.2.1	−1.95 *	−0.08	−0.47	−0.43	60S ribosomal protein L13a-like protein
Solyc12g098330.1.1	−2.83 *	−0.08	−0.24	−0.25	60S ribosomal protein L13a-like protein
Solyc06g073300.1.1	−1.70 *	−0.57	−0.62	0.23	60S ribosomal protein L27
Solyc03g112360.1.1	−1.75 *	−0.56	−0.64	−0.66	60S ribosomal protein L27A
Solyc07g008370.2.1	−1.68 *	0.11	−0.62	0.01	60S ribosomal protein L7
Solyc06g072570.2.1	−2.16 *	0.01	−0.54	−0.14	Ubiquitin-conjugating enzyme E2 1
Solyc12g062250.1.1	−1.61 *	−0.16	−0.34	0.20	5′-AMP-activated protein kinase
Solyc04g008810.2.1	−0.49	−0.91 *	0.00	−0.20	40S ribosomal protein S26
Solyc04g009770.2.1	0.43	−0.81 *	0.67	−0.14	DNAJ chaperone
Solyc09g098280.2.1	−0.57	−1.31 *	−0.08	0.03	Eukaryotic translation initiation factor 3 subunit 3
Solyc02g064670.1.1	−0.37	−1.08 *	−0.03	0.11	Ribosomal protein L26-like 1
Solyc05g053780.2.1	−0.18	−1.03 *	−0.56	−0.43	RNA-binding protein-like protein
Solyc06g008130.2.1	0.35	−0.99 *	0.07	−0.28	SET domain protein SUVR2
Solyc10g080180.1.1	0.08	−0.83 *	0.37	0.13	U3 small nucleolar RNA-associated protein 18
Solyc04g010030.1.1	0.23	−0.83 *	0.71	0.08	Thioredoxin-like protein 1
Solyc12g056220.1.1	0.87	0.93 *	−0.59	−0.38	Aquaporin
Solyc09g083380.2.1	−0.90	−1.04	−0.93 *	−0.50	Histone H1
Solyc11g007930.1.1	−0.68	−0.76	−1.80 *	−0.13	Histone H2B
Solyc10g081030.1.1	−0.38	−0.46	−0.86 *	−0.68	Nascent polypeptide-associated complex alpha subunit
Solyc08g016420.2.1	−0.37	−0.70	−2.14 *	−0.46	Prefoldin subunit 6
Solyc03g121310.2.1	0.07	−0.81	−1.03 *	0.01	RWD domain-containing protein
Solyc03g119380.1.1	−0.08	−0.28	−0.06	−0.79 *	50S ribosomal protein L14
Solyc09g091740.2.1	−0.01	0.10	0.29	−0.97 *	60S ribosomal protein L13a-like protein
Solyc01g098000.2.1	−0.25	0.06	−0.65	−0.72 *	Elongation factor-like protein
Solyc01g109660.2.1	0.05	−0.63	−0.56	−0.68 *	Glycine-rich RNA-binding protein
Solyc05g051290.2.1	0.23	−0.23	−0.41	−0.74 *	High-mobility group family
Solyc02g083470.2.1	−0.02	−1.08	−0.11	−1.08 *	Pre-rRNA-processing protein ESF1
Solyc11g005170.1.1	−0.25	−0.49	0.25	−1.00 *	RNA-splicing factor
Solyc01g095050.2.1	−0.39	−0.63	−2.00 *	−0.44	Negatively light-regulated protein
Solyc12g014420.1.1	0.11	0.20	0.76	0.77 *	Glucan endo-1 3-beta-glucosidase A6
Proteins with consistent HS responses					
Solyc06g069220.1.1	4.60 *	0.82 *	5.98 *	1.10 *	Aspartic proteinase nepenthesin I
Solyc03g098710.1.1	2.36 *	1.10 *	1.25 *	1.01 *	Kunitz-type proteinase inhibitor A4
Solyc00g187050.2.1	1.75 *	1.15 *	1.99 *	1.07 *	Leucyl aminopeptidase
Solyc12g010020.1.1	2.11 *	1.30 *	2.18 *	1.09 *	Leucyl aminopeptidase
Solyc08g079910.1.1	1.27 *	0.71 *	2.89 *	1.07 *	Subtilisin-like protease
Solyc07g064600.2.1	2.95 *	0.81 *	2.23 *	1.10 *	Endoribonuclease L-PSP family protein
Solyc08g076970.2.1	1.82 *	0.91 *	2.34 *	1.52 *	Acetylornithine deacetylase
Solyc01g066460.2.1	0.90 *	3.68 *	1.77 *	1.39 *	Epoxide hydrolase
Solyc09g008670.2.1	1.69 *	0.90 *	2.91 *	0.73 *	Threonine ammonia-lyase biosynthetic
Solyc01g010390.2.1	3.03 *	0.82 *	3.56 *	1.31 *	Beta-glucosidase
Solyc12g094460.1.1	1.30 *	0.73 *	2.43 *	1.40 *	Laccase-2
Solyc04g079890.2.1	3.69 *	1.17 *	4.23 *	1.41 *	Thaumatin-like protein

* Heat stress induced differentially abundant proteins (DAPs), which were defined by a fold change greater than two standard deviations (SDs) and using an FDR-adjusted *p*-value threshold of ≤0.05 in the specific cells within the same variety. ^1,2^ Tomato varieties, BV for ‘Black Vernissage’, MT for ‘Micro-Tom’. ^3^ PMCs: pollen mother cells.

## Data Availability

The mass spectrometry proteomics data have been deposited to the ProteomeXchange Consortium via the PRIDE partner repository with the dataset identifiers PXD060801 and 10.6019/PXD060801 under the project name of ‘Heat stress induced proteomes in tomato pollen mother cells and microspores’.

## Supplementary Materials

The following supporting information can be downloaded at https://www.mdpi.com/article/10.3390/proteomes13020013/s1. Table S1: The heat stress-induced pollen mother cell proteomes; Table S2: Meiotic processes enriched for pollen mother cells; Table S3: The heat stress-induced free microspore proteomes; Table S4: Biological processes enriched for microspore cells; Table S5: STRING MCL clusters for HS-upregulated proteins in pollen mother cells of ‘Black Vernissage’; Table S6: STRING MCL clusters for HS-downregulated proteins in pollen mother cells of ‘Black Vernissage’; Table S7: STRING MCL clusters for HS-upregulated proteins in pollen mother cells of ‘Micro-Tom’; Table S8: STRING MCL clusters for HS-downregulated proteins in pollen mother cells of ‘Micro-Tom’; Table S9: STRING MCL clusters for HS-upregulated proteins in microspores of ‘Black Vernissage’; Table S10: STRING MCL clusters for HS-downregulated proteins in microspores of ‘Black Vernissage’; Table S11: STRING MCL clusters for HS-upregulated proteins in microspores of ‘Micro-Tom’; Table S12: STRING MCL clusters for HS-downregulated proteins in microspores of ‘Micro-Tom’.

## Declaration

The mention of tomato varieties and trade names are used in this publication solely to provide specific information. The mention of a trade name does not constitute a warranty or an endorsement of the product by the U.S. Department of Agriculture to the exclusion of other products not mentioned.
